# Aqueous humour cytokine profiles in retinal vein occlusion: a comparative study of acute, chronic recurrent, and control eyes

**DOI:** 10.1007/s00417-026-07152-0

**Published:** 2026-02-21

**Authors:** Satoru Inoda, Hidenori Takahashi, Chihiro Mayama, Xue Tan, Yoko Nomura, Yujiro Fujino, Hidetoshi Kawashima, Toshikatsu Kaburaki, Yasuo Yanagi

**Affiliations:** 1 Department of Ophthalmology, Jichi Medical University, 3311-1 Yakushiji, Shimotsuke-shi, Tochigi 329-0498 Japan; 2 Department of Ophthalmology, Graduate School of Medicine, the University of Tokyo, 7-3- 1 Hongo, Bunkyo-ku, Tokyo, 113-8655 Japan; 3 Japan Community Healthcare Organization Tokyo Shinjuku Medical Center, 5-1 Tsukudocho, Shinjuku-ku, Tokyo, 162-8543 Japan; 4 Center for Cyber Medicine Research, University of Tsukuba, 5-1-1 Tennodai, Tsukuba, Ibaraki 305-8575 Japan; 5 Medical Retina, Singapore National Eye Centre, 11 Third Hospital Ave, Singapore, 168751 Singapore; 6 Medical Retina, Singapore Eye Research Institute, 11 Third Hospital Ave, Singapore, 168751 Singapore; 7 The Ophthalmology & Visual Sciences Academic Clinical Program, Duke-NUS Medical School, National University of Singapore, 8 College Road, Singapore, 169857 Singapore

**Keywords:** Inflammatory cytokines, Aqueous humour, Cystic macular oedema, Branch retinal vein occlusion, Central retinal vein occlusion

## Abstract

**Purpose:**

This study aimed to characterize inflammatory cytokine profiles in recurrent retinal vein occlusion (RVO) and to describe subtype- and phase-specific patterns relative to controls.

**Methods:**

This was retrospective study conducted from September 2010 to September 2022 at the Japan Community Healthcare Organization Tokyo Shinjuku Medical Center and Jichi Medical University. Aqueous humour (AqH) was collected immediately before intravitreal bevacizumab, ranibizumab or aflibercept injections from patients with RVO. We included an acute group (RVO onset within 3 months) and a recurrent group (patients with disease duration of ≥ 1 year). Using a multiplex cytokine assay, we measured the concentrations of nine cytokines and VEGF. The results were compared with controls after adjusting for sex, age, axial length, and posterior vitreous detachment.

**Results:**

AqH samples from 30 acute branch RVO (BRVO), 31 recurrent BRVO, 25 acute central RVO (CRVO), 20 recurrent CRVO, and 81 cataract surgery eyes without retinal disease (as controls) were used. In recurrent BRVO, MCP-1, CXCL13, and CCL11 levels were significantly lower than those in control eyes (all, *P* < 0.05). Similarly, in recurrent CRVO, MCP-1, CXCL12, CXCL13, CCL11, and CXCL1 levels were significantly reduced compared with controls (all, *P* < 0.05), whereas IL-6 levels were significantly elevated (*P* < 0.05).　In acute RVO, cytokine alterations compared with controls were limited. Acute BRVO showed decreased MCP-1, CXCL13, and CCL11 levels (all, *P* < 0.05), while acute CRVO exhibited increased IL-6 and IP-10 levels (all, *P* < 0.05).

**Conclusions:**

Inflammatory cytokine profiles in recurrent RVO differ between BRVO and CRVO and are characterized by reduced levels of multiple inflammatory cytokines compared with controls. These findings highlight distinct inflammatory features of recurrent RVO and provide a basis for future studies investigating disease progression and treatment resistance.

**Trial registration:**

UMIN000020718 https://center6.umin.ac.jp/cgi-open-bin/icdr_e/ctr_his_list.cgi?recptno=R000023907.

**Key messages:**

**What is known:**

- While anti- endothelial growth factor (VEGF) therapy is effective for treating cystic macular oedema (CME) secondary to retinal vein occlusion (RVO), the condition is not confined to the acute phase. The frequent chronicity and recurrence of CME imply that other pathological factors, beyond VEGF, are involved. Furthermore, preceding research on acute RVO has documented increased levels of inflammatory cytokines, including IL-6, IL-8, and MCP-1.

**What is new:**

- Inflammatory cytokine profiles differ between branch RVO and central RVO in both acute and recurrent cases when each is explained relative to controls using multivariable-adjusted analyses. In particular, recurrent RVO is characterized by reduced levels of several inflammatory cytokines compared with controls, indicating a distinct inflammatory profile in the recurrent phase and providing insights into disease progression.

**Supplementary Information:**

The online version contains supplementary material available at 10.1007/s00417-026-07152-0.

## Introduction

Cystic macular oedema (CME) is among the most common causes of vision loss in eyes with central retinal vein occlusion (CRVO) and branch retinal vein occlusion (BRVO). Intravitreal injection of anti-vascular endothelial growth factor (VEGF) antibodies, such as aflibercept, ranibizumab, or bevacizumab, improves CME in patients with RVO [1] .However, most patients need multiple injections because of recurrent CME [2] .Using pro re nata (PRN) treatment protocol, even in the fourth year of treatment, 17 of 34 patients with CME associated with BRVO reportedly needed an average of 3.2 injections of intravitreal anti-VEGF drugs [3].This highlights the involvement of VEGF-independent pathways and other inflammatory or pro-angiogenic factors in the persistent pathology. Consequently, there is growing interest in exploring novel therapeutic strategies that can target these alternative mechanisms. Receptor tyrosine kinase inhibitors (RTKi), capable of modulating multiple signalling pathways, represent a promising class of agents for addressing the complex and multifactorial nature of chronic RVO.

Although VEGF is the key player in the pathogenesis of CME in RVO, mechanisms other than VEGF contribute to CME due to RVO. Noma et al. reported the elevation of both IL-6 and VEGF levels in RVO; importantly, both of these were correlated with ischaemic area size in acute BRVO [4]. Shimura et al. reported that the vitreous level of IL-6 was correlated with both improvement in best corrected visual acuity (BCVA) and decrease in foveal thickness after pars plana vitrectomy with arteriovenous sheathotomy in patients with BRVO associated with CME [5]. Fonollosa et al. reported that IL-8 and MCP-1 levels were elevated in the vitreous humour of patients with BRVO associated with CME [6]. Other angiogenic and inflammatory cytokines, for example sICAM-1, IL-12, PIGF, sVEGFR-1, sVEGFR-2, IL-13, and PDGF-AA, are elevated in the aqueous humour of patients with acute CRVO [7]. Thus, it has become clear that inflammatory cytokines have an important role in CME in eyes with RVO besides VEGF.

Critically, all previous studies on RVO cytokine profiles have focused solely on the acute phase of the disease, with sampling of aqueous or vitreous humour conducted within 4 weeks to 4.7 ± 2.2 months of onset, leaving chronic recurrent disease unexplored. Thus, the cytokine profiles of patients with chronic recurrent CME are not known. To address this gap, we examined the levels of multiple inflammatory cytokines in the aqueous humour of patients with recurrent RVO and compared them with those of control eyes, while also describing cytokine alterations observed in acute RVO for contextual reference. By characterizing inflammatory profiles in recurrent RVO, this study aims to provide insights into inflammatory involvement in recurrent disease and to establish a foundation for future studies investigating disease progression and treatment resistance in RVO.

## Methods

### Study design

This study is a retrospective study in which aqueous humour was collected, and cytokine measurements were performed after all the samples were collected. Institutional review board approval was obtained from the Japan Community Healthcare Organization (JCHO) Tokyo Shinjuku Medical Center and Jichi Medical University. Written informed consent was obtained from all patients.

### Materials

**Fig. 1:**
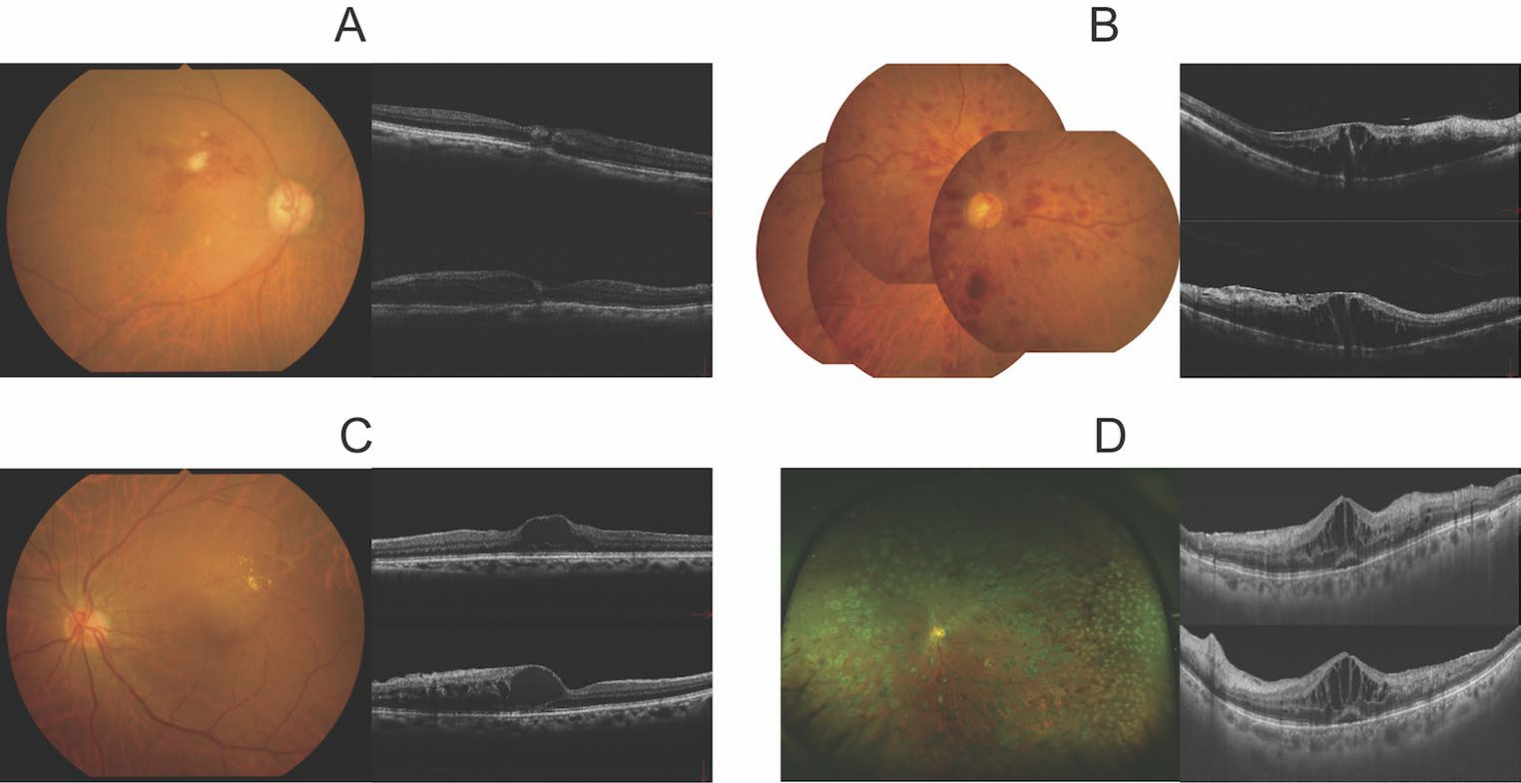
Representative cases for each disease type. **A**. Acute BRVO: Fundus photography is shown on the left, and OCT image on the right. **B**. Acute CRVO: Fundus photography is shown on the left, and OCT image on the right. **C**. Recurrent BRVO: Fundus photography is shown on the left, and OCT image on the right. **D**. Recurrent CRVO: Fundus photography is shown on the left, and OCT image on the right

Patients with RVO onset within 3 months and those with recurrence of CME after 1 year or more were included in this study. CME was evaluated using vertical and horizontal optical coherence tomography scans. Samples were collected from 106 eyes of 106 patients who were recruited consecutively at JCHO Tokyo Shinjuku Medical Center and Jichi Medical University from September 2010 to September 2022. Data of 30 acute BRVO, 31 recurrent BRVO, 25 acute CRVO, and 20 recurrent CRVO patients were analysed. For the purposes of analysis, hemi-central retinal vein occlusion (hemi-CRVO) cases were classified as CRVO. Images of each disease subtype are presented along with the measured cytokine results (Fig. 1; Table 1) Treatment-naïve patients with disease onset within 3 months were classified as acute. Patients with disease duration of ≥ 1 year were classified as recurrent. Patients who had received periocular steroid or anti-VEGF injections within 4 months or had undergone retinal photocoagulation were excluded. Patients were treated on a PRN regimen, and thus the recurrent group comprised those who had been in remission for > 4 months. No patients received intraocular steroid injections. All CRVO patients were non-ischaemic as judged by fluorescein angiography. In total, 81 cataract surgery eyes were used as controls, excluding those with existing retinal disease or those taking immunosuppressants or oral steroids.

**Table 1:** Representative cases and cytokine levels

	Acute BRVO	Recurrent BRVO	Acute CRVO	Recurrent CRVO
IL-6 (pg/ml)	12	25.1	115.8	35.6
IL-10 (pg/ml)	0.49	1.67	0.16	0.73
VEGF (pg/ml)	136.3	88.4	55	43.7
IP-10 (pg/ml)	11.2	64.1	132.2	315.7
MCP-1 (pg/ml)	220.7	279.1	439.1	586.7
CCL11 (pg/ml)	0.43	7.33	2.38	1.45
CXCL1 (pg/ml)	1.89	8.03	0.87	7.06
CXCL12 (pg/ml)	1.7	187.5	288.7	25.7
CXCL13 (pg/ml)	0.05	3.34	2.34	1.91

*BRVO* Branch retinal vein occlusion; *CRVO* Central retinal vein occlusion

IL-6, interleukin − 6; IL-10, interleukin; IP-10, interferon-γ-inducible protein 10; VEGF, vascular endothelial growth factor; MCP-1, monocyte chemoattractant protein-1;CXCL1, C-X-C motif chemokine ligand 1; CXCL12, C-X-C motif chemokine ligand 12; CXCL13, C-X-C motif chemokine ligand 13; CCL11, C-C motif chemokine ligand 11,

### Sample collection

For RVO patients, approximately 0.2 mL of aqueous humour was collected with a 30-gauge needle just before intravitreal bevacizumab, ranibizumab or aflibercept injection. In this retrospective study, the choice of anti-VEGF agent was left to the discretion of the treating physician and was also influenced by drug availability at the time of treatment, including the year of approval. For controls, at the beginning of cataract surgery, a sample of undiluted aqueous humour (usually, about 0.2 mL) was aspirated manually into a disposable syringe, immediately transferred to a sterile tube, and stored at -80 °C until assay.

### Measurement of cytokines

The concentrations of the following cytokines were analysed in aqueous humour samples collected September 2010 to September 2022 using Bio-Plex-Pro™ Human Chemokine Assays (Bio-Rad, California, U.S.A) according to the manufacturer’s instructions; interleukin − 6 (IL-6), IL-10, interferon-γ-inducible protein 10 (IP-10), monocyte chemoattractant protein-1 (MCP-1), C-X-C motif chemokine ligand 12 (CXCL12), CXCL13, C-C motif chemokine ligand 11 (CCL11), CXCL1, and vascular endothelial growth factor (VEGF). The detection limits were 0.1, 0.9, 1.1, 0.10, 10.3, 0.1, 0.7, 4.2 and 5.0 pg/mL for IL-6, IL-10, IP-10, MCP-1, CXCL12, CXCL13, CCL11, CXCL1, and VEGF respectively. We selected these cytokines based on recent literature and our recent research [8–11]. Additionally, we summarized their basic biological roles in a Supplemental Table 1.

Log-transformed concentrations were compared with those of controls by using multiple linear regressions after adjusting for sex, age, axial length, and posterior vitreous detachment (PVD). Measurements were performed twice for each sample and the average was calculated and used for the analysis.

### Ultrasonography

Axial length was examined by A-mode ultrasonography (UD-6000; TOMEY Corp., Aichi, Japan). PVD was examined by B-mode ultrasonography (UD-6000), similar to the published work of previous investigations [12]. Briefly, the mobility of the posterior vitreous during ocular saccades was examined using the ‘through-the-lid contact’ technique. If the posterior vitreous was detached from the retinal surface and motile with eye movements, the eyes were categorized as having complete PVD; otherwise, the eyes were categorized as without PVD (including eyes with partial PVD and no PVD).

### Statistical analysis

Statistical analysis was performed using JMP Pro software version 17.2.0 (SAS Institute, Cary, NC). Patient demographics were analysed using a Chi-squared test and Dunnett’s test. Regression model and adjustment were corrected by “standard least square” multiple regression analysis. When concentrations were compared between the RVO and control groups, the associations between baseline characteristic factors, such as age, sex, axial length, and the presence or absence of PVD, were corrected by multiple regression analysis [13, 14]. 

Statistical models using log-transformed concentrations were used because of the skewed distribution of this variable. One patient in the acute CRVO group presented with bilateral involvement. Given the small number, this was treated as a single observation, and its inclusion was considered to have a negligible effect on the statistical results.

## Results

### Demographic characteristics of the patients

Table 2 summarizes the demographic characteristics of the patients and controls. The mean age of the acute BRVO, recurrent BRVO, acute CRVO, recurrent CRVO, and control groups was 66.2 (standard deviation [SD]) ± 12.9, 71.8 ± 8.6, 70.8 ± 15.2, 71.4 ± 12.2, and 74.7 ± 6.8 years old. The mean age of acute BRVO was significantly younger than that of control (*P* = 0.008). Log MAR VA was significantly worse in early and late CRVO cases compared with control (*P* = 0.017, < 0.001, respectively), and CMT was thicker in all RVO cases than in controls (*P*< 0.0001, all).

**Table 2:** Demographic characteristics of patients with RVO

	Control	Acute BRVO		Recurrent BRVO		Acute CRVO		Recurrent CRVO	*P*-value
*N*, eyes	81	30		31		25		20	
PVD+ (*n* (%))*	47 (60)	10 (36)		18(60)		16 (64)		13 (65.0)	0.16
Male (*n* (%))*	36 (44)	15 (50)		13 (42)		17 (68)		14 (70.0)	0.085
Hypertension, (*n* (%))*		26		29		19		16	0.53
Diabetic Mellitus, (*n* (%))*		0		2		5		1	0.11
Hyperlipemia (*n* (%))*		2		1		5		1	0.83
Lens status, aphakia (*n* (%))*		5		9		5		5	0.34
			*P*-value		*P*-value		*P*-value		*P*-value
Age (years, mean ± SD) ^†^	74.7 ± 6.8	66.2 ± 12.9	**0.015**	71.8 ± 10.0	0.99	70.8 ± 15.2	0.43	71.4 ± 12.2	0.99
Axial length (mm, mean ± SD) ^†^	23.6 ± 1.2	23.8 ± 1.5	0.99	23.6 ± 1.5	0.99	24.0 ± 1.5	0.99	23.6 ± 1.2	0.99
VA (log MAR, mean ± SD) ^†^	0.29 ± 0.36	0.36 ± 0.33	0.99	0.28 ± 0.28	0.99	0.64 ± 0.54	**0.017**	0.79 ± 0.74	**0.0033**
CMT (µm, mean ± SD) ^†^	228 ± 46	556 ± 202	**< 0.0001**	507 ± 126	**< 0.0001**	646 ± 281	**< 0.0001**	580 ± 333	**< 0.0001**

*BRVO* Branch retinal vein occlusion; *CMT* Central macular thickness; *CRVO* Central retinal vein occlusion; *MAR* Minimal angle of resolution; *PVD* Posterior vitreous detachment; *SD* Standard deviation; *VA* Visual acuity

*Pearson’s chi-square test

^†^vs. control; Dunnett test

In the BRVO group, macular BRVO cases accounted for 23 and 17 patients in the acute and recurrent subgroups, respectively.

Five patients underwent PC more than 4 months ago. These cases consisted of 2 with recurrent BRVO, and 3 with recurrent CRVO.

### Cytokine concentrations in the aqueous humour of RVO patients

**Fig. 2:**
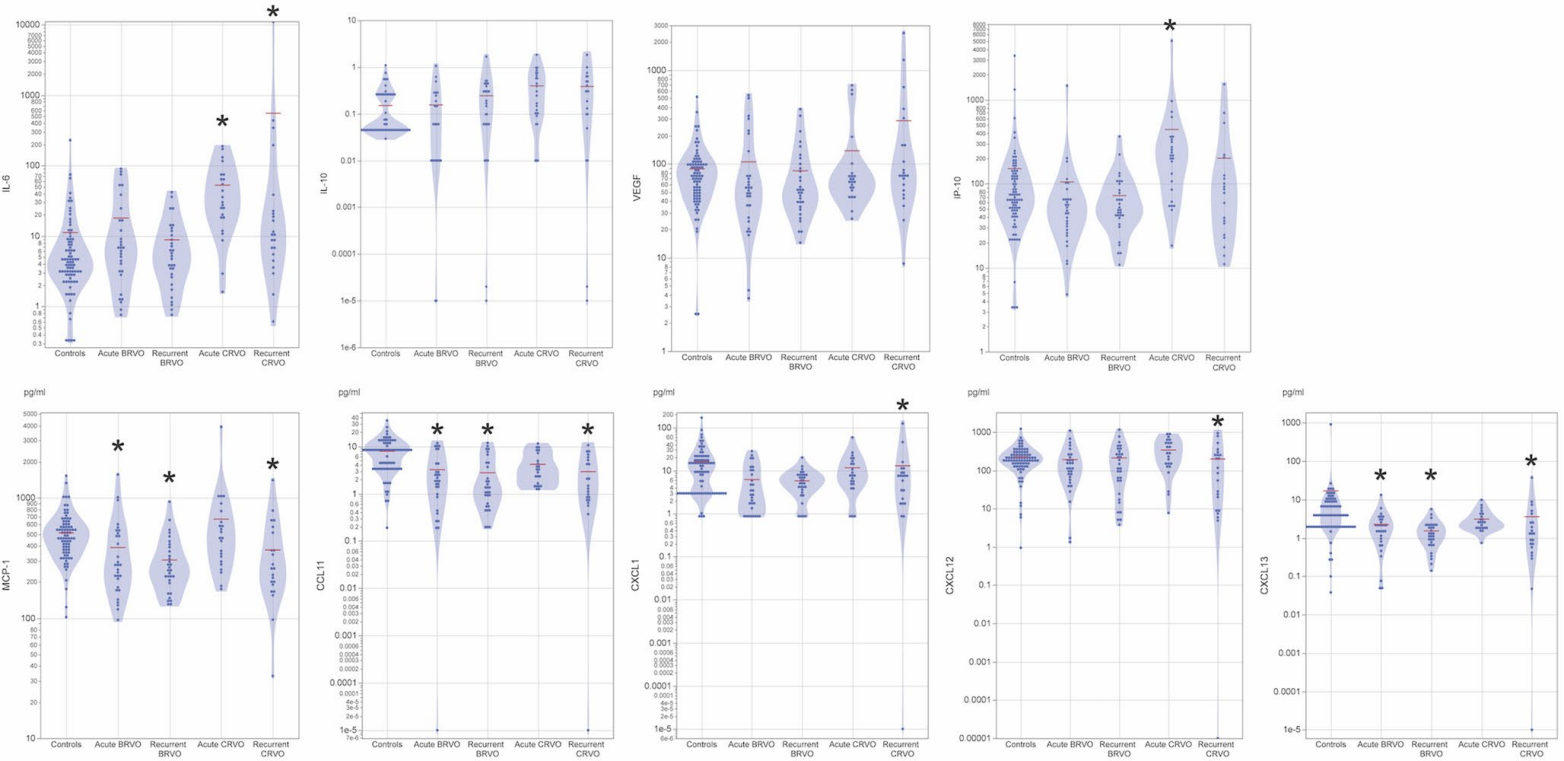
Cytokine levels in patients with acute and recurrent retinal vein occlusion, and control subjects (unadjusted raw data). Red bars indicate mean cytokine concentrations. Comparisons were performed using least-squares means adjusted for age, sex, axial length, and posterior vitreous detachment, with the control group as the reference (Dunnett’s test). **A**. IL-6 levels in patients with acute, recurrent retinal vein occlusion (RVO), and control subjects. IL-6 levels were significantly higher in acute CRVO (*P* < 0.0001) and recurrent CRVO (*P* = 0.0099) compared with controls. No significant differences were observed in acute BRVO or recurrent BRVO. **B**. IL-10 levels in patients with acute, recurrent retinal vein occlusion, and control subjects. No significant differences in IL-10 levels were observed among the RVO groups compared with controls. **C**. VEGF levels in patients with acute, recurrent retinal vein occlusion, and control subjects. No significant differences in VEGF levels were observed between any RVO group and controls. **D**. IP-10 levels in patients with acute, recurrent retinal vein occlusion, and control subjects. IP-10 levels were significantly higher in acute CRVO compared with controls (*P* < 0.0001). **E**. MCP-1 levels in patients with acute, recurrent retinal vein occlusion, and control subjects. MCP-1 levels were significantly lower in acute BRVO (*P* = 0.0266), recurrent BRVO (*P* < 0.0001), and recurrent CRVO (*P* = 0.0012) compared with controls. **F**. CCL11 levels in patients with acute, recurrent retinal vein occlusion, and control subjects. CCL11 levels were significantly lower in acute BRVO (*P* = 0.0007), recurrent BRVO (*P* = 0.0002), and recurrent CRVO (*P* < 0.0001) compared with controls. **G**. CXCL1 levels in patients with acute, recurrent retinal vein occlusion, and control subjects. CXCL1 levels were significantly lower in recurrent CRVO compared with controls (*P* = 0.0196). **H**. CXCL12 levels in patients with acute, recurrent retinal vein occlusion, and control subjects. CXCL12 levels were significantly lower in recurrent CRVO compared with controls (*P* < 0.0001). **I**. CXCL13 levels in patients with acute, recurrent retinal vein occlusion, and control subjects. CXCL13 levels were significantly lower in acute BRVO (*P* = 0.00013), recurrent BRVO (*P* < 0.0001), and recurrent CRVO (*P* < 0.0001) compared with controls. BRVO, branch retinal vein occlusion; CRVO, central retinal vein occlusion. The vertical axis of the graph is logarithmically scaled. * Indicates a statistically significant difference compared to the control group after adjustment for covariates (age, sex, axial length, and the presence or absence of PVD), with *P* < 0.05

The concentrations of cytokines in the aqueous humour of controls and RVO patients are shown in Fig. 2. Overall, cytokine alterations compared with controls were more prominent in CRVO than in BRVO. In acute BRVO, no cytokines were significantly higher compared with controls. However, MCP-1 (mean ± SD, 386.4 ± 318.2 vs. 512.4 ± 241.0, *P* = 0.0266), CXCL13 (2.23 ± 2.46 vs. 16.84 ± 100.87, *P* = 0.00013), and CCL11 (3.21 ± 3.49 vs. 7.90 ± 5.76, *P* = 0.0007) levels were significantly lower than those in controls. In recurrent BRVO, several inflammatory cytokines were significantly reduced compared with controls, including MCP-1 (304.3 ± 175.4 vs. 512.4 ± 241.0, *P* < 0.0001), CXCL13 (1.52 ± 1.19 vs. 16.84 ± 100.87, *P* < 0.0001), and CCL11 (2.77 ± 3.25 vs. 7.90 ± 5.76, *P* = 0.0002). No cytokines were significantly elevated in recurrent BRVO.

The change in the aqueous humour cytokine concentrations was more drastic in the CRVO group than in the BRVO group. In acute CRVO, IL-6 (73.7 ± 94.8 vs. 11.18 ± 27.58, *P* < 0.0001) and IP-10 (444.0 ± 986.2 vs. 152.1 ± 396.3, *P* < 0.0001) levels were significantly higher than those in controls.

In recurrent CRVO, multiple cytokines were significantly reduced compared with controls, including MCP-1 (360.2 ± 313.1 vs. 512.4 ± 241.0, *P* = 0.0012), CXCL12 (191.6 ± 267.5 vs. 225.3 ± 170.4, *P* < 0.0001), CXCL13 (1.68 ± 1.87 vs. 16.84 ± 100.87, *P* < 0.0001), CCL11 (2.80 ± 3.14 vs. 7.90 ± 5.76, *P* < 0.0001), and CXCL1 (11.96 ± 26.72 vs. 16.25 ± 22.92, *P* = 0.0196). In contrast, IL-6 levels were significantly elevated in recurrent CRVO compared with controls (574.7 ± 2444.1 vs. 11.18 ± 27.58, *P* = 0.0099). No significant differences in VEGF levels were observed in either acute or recurrent CRVO compared with controls.

Four patients with recurrent BRVO and one patient with recurrent CRVO received sub-Tenon’s triamcinolone acetonide injection (STTA) more than four months prior to the analysis. Focusing on the concentration of the inflammatory cytokine IL-6, the levels in the four recurrent BRVO patients fell within the 95th percentile. Conversely, the concentration in the patient with recurrent CRVO was markedly elevated, exceeding the 95th percentile. Similarly, twelve patients each with recurrent BRVO and recurrent CRVO had received anti-VEGF injections more than four months prior to sampling. There was no significant difference in VEGF concentration based on the history of prior anti-VEGF agent administration in either disease type (*P* = 0.44 for recurrent BRVO and *P* = 0.65 for recurrent CRVO).

We did not find any correlation between cytokine concentration and CME.

## Discussion

To the best of our knowledge, this is the first study to characterize inflammatory cytokine profiles in recurrent retinal vein occlusion (RVO) using a control-referenced, multivariable-adjusted analytical framework. By adjusting for age, sex, axial length, and posterior vitreous detachment, we identified distinct cytokine alterations in recurrent RVO compared with control eyes, with differences observed between branch and central RVO. Several inflammatory cytokines, including MCP-1, CXCL12, CXCL13, CCL11, and CXCL1, were significantly reduced in recurrent RVO subtypes, whereas cytokine elevations were limited and disease subtype–specific.

In acute CRVO, selected inflammatory cytokines, including IL-6 and IP-10, were elevated compared with controls, consistent with an active inflammatory state in the acute phase. Notably, cytokine regulation differed between CRVO and BRVO, indicating heterogeneity in inflammatory involvement according to disease subtype. Although direct temporal comparisons between acute and recurrent disease were not performed, the distinct cytokine profiles observed in recurrent RVO, when interpreted in the context of previous studies focusing on acute RVO, may suggest differences in inflammatory involvement across disease stages. Recurrent RVO represents a clinically complex condition influenced by long disease duration and repeated therapeutic interventions, which may be associated with alterations in inflammatory and angiogenic signalling pathways. The altered profile in chronic recurrent RVO, possibly associated with long disease duration and repeated treatments, may be consistent with involvement of alternative pro-angiogenic and inflammatory pathways, possibly involving growth factors like PDGF and FGF and their respective receptor tyrosine kinases (RTKs). Understanding these pathway-specific mechanistic contexts will be crucial for developing targeted therapeutic strategies that can effectively interrupt the vicious cycle of inflammation and angiogenesis driving chronic recurrent RVO.

### Regulation of cytokines in acute and recurrent RVO

IL-6 and IP-10 were increased in acute CRVO, as demonstrated previously [15–17]. We did not find upregulation of any cytokines in acute BRVO in this group, which contrasts to previous studies [18]. For example, IL-6 has been reported to be elevated in acute BRVO and are known to be associated with the severity of CME and resistance to anti-VEGF drugs [19]. We believe that this apparent discrepancy is attributable to the fact that most patients in the present study had less severe BRVO compared with previous studies, including the exclusion of severe cases such as those with marked vitreous hemorrhage. This is presumably because referring physicians tend to refer patients to retinal specialists earlier due to the availability of anti-VEGF drugs over the past few years. In support of this, the presenting VA of patients with BRVO was relatively good compared with previous studies [20]. Additionally, the present results clearly showed that inflammatory cytokines are regulated differently between acute and recurrent RVO. No cytokines were increased in recurrent RVO excluding IL-6, and rather, several inflammatory cytokines, namely MCP-1, CXCL12, CXCL13, CCL11, and CXCL1 were decreased in recurrent RVO patients. The mechanism underlying this downregulation is not clear. Because most patients were on several anti-VEGF injections, this might have contributed to the resolution of the parainflammation occurring in the ageing normal retina [21].

Recurrent macular oedema is a clinically important challenge. Previous reports have also indicated the efficacy of triamcinolone acetonide for CME associated with RVO [22–25]. However, it has also been reported that the efficacy of triamcinolone acetonide is lower in cases where a significant amount of time has passed since onset and anti-VEGF drugs have become less effective [26]. Moreover, to directly address the link between cytokine changes and recurrence, we have previously reported that the combined therapy of anti-VEGF and STTA prolonged the time to recurrence of macular oedema in 60% of patients with recurrent RVO [11]. In those cases exhibiting this prolongation, a significant upregulation of IL-1α and a significant downregulation of IL-5, IL-6, and galectin-1 were observed. These observations corroborate our present findings, implying that the intraocular milieu undergoes alterations recurrent RVO, a form of chronic disease activity, with the passage of time compared to the acute stage, potentially leading to the inadequacy of VEGF or triamcinolone acetonide monotherapy and suggesting the potential efficacy of adjunctive triamcinolone in some patients. Retinal degeneration or atrophy occurring in long-standing disease could also explain the decreased expression. Indeed, the reduction in cytokine levels exhibited a similar trend between BRVO and CRVO with varying extents of occlusion, with CRVO demonstrating a decrease in a broader range of cytokines. Interestingly, despite the absence of significant VEGF elevation compared with controls, anti-VEGF therapy may still provide clinical benefit in chronic CRVO, potentially through mechanisms not captured by single-timepoint aqueous measurements.

### Possible roles of IL-6, MCP-1 and VEGF loop

It is generally accepted that venous occlusion in RVO induces hypoxia, which stimulates the expression of IL-6 subsequently inducing the expression of various other inflammatory cytokines, including VEGF [27]. In addition, VEGF directly increases the production of IL-6 from human peripheral blood mononuclear cells [28]. These cytokine loops are significantly correlated with the nonperfusion area of BRVO and the severity of CME in acute BRVO [7]. MCP-1 is known to play an important role in acute RVO through its interaction with VEGF, as reported in previous studies [9, 18]. Although our analysis did not show a significant elevation of MCP-1 levels in acute CRVO compared with controls, these findings support the involvement of MCP-1–VEGF–mediated inflammatory signaling in the acute phase of RVO.

### Other cytokines—IP-10, CXCL1, CXCL12, CXCL13, and CCL11

Our results indicate that selected pro- and anti-angiogenic/inflammatory cytokines show altered expression patterns in RVO, depending on disease subtype and phase. IP-10 is an anti-angiogenic factor and also modulates inflammation [29]. IP-10 levels in the aqueous humour were also higher in RVO patients in a previous report [9]. In contrast to IP-10, CXCL12, also known as stromal cell-derived factor 1, is considered as pro-angiogenic [17]. Intravitreal CXCL12 levels in RVO patients with iris neovascularisation were reportedly elevated, but not in those without iris neovascularisation. Consistent with these reports, CXCL12 levels were not elevated in recurrent CRVO or BRVO compared with controls in the present study. The role of CXCL1 and CXCL13 are less clear in the context of RVO compared with other cytokines. CXCL1 is known to be critical for retinal leukocyte recruitment and ischemia/reperfusion induced retinopathy and plays a critical role in the pathogenesis of hypertensive retinopathy [30, 31]. CXCL-1 is also kwon to alter the blood-retinal barrier in diabetic retinopathy [32]. CXCL13 is a B lymphocyte chemoattractant and a B cell-specific chemokine; therefore, it is possible that B lymphocytes play some role in the CRVO inflammatory cascade [33]. In the eyes of healthy subjects, CXCL13 levels correlate with subfoveal choroidal thickness [34]. CCL11 is a pro-angiogenic cytokine that activates VEGF receptor 2 signalling via binding to the specific receptor CCR3 [35]. CCL11 levels were significantly higher in PDR patients, and this cytokine may have an angiogenic effect on retinal neovascular diseases such as PDR [36]. Taken together, these findings suggest that, in addition to the MCP-1–VEGF loop, multiple pro- and anti-angiogenic cytokines may contribute to the inflammatory environment in RVO. However, their relative involvement may differ between acute and recurrent disease, warranting further investigation.

### Clinical implications and future directions

As we reported previously, anti-VEGF therapy reduces inflammatory cytokine levels in the aqueous humour of eyes with age-related macular degeneration [37]. Our results suggest that inflammatory cytokines may play a less prominent role in recurrent RVO with CME. As has been demonstrated in previous studies, chronic CME is not caused by inflammation alone. Microaneurysm formation is observed in RVO cases at only a few months after disease onset, and chronic leakage from microaneurysms also plays an important role in the pathogenesis of chronic CME in the setting of CRVO/BRVO. In support of this, focal laser photocoagulation, which partly occludes microaneurysms, was shown to be effective in reducing central retinal thickness in cases with CME due to BRVO and CRVO (although there was no benefit of laser photocoagulation for visual acuity gain in cases with CRVO). Hence, these findings indicate that treatment strategies targeting mechanisms beyond inflammation may be required in recurrent RVO. Indeed, a recent study clearly demonstrated that when microaneurysms are present, direct photocoagulation is effective for reducing chronic CME (and improving visual acuity) due to RVOs [38].

### Limitations

This study had several limitations. First, the subjects were from two institutions, and a relatively small number of subjects were analysed. Reduced levels of cytokines may be explained by possible retinal degeneration, and it may be related to the time to recurrence of CME and/or the frequency of recurrence. In this study, the number of subjects was too small to examine this interpretation. A study with a larger number of subjects may clarify this interpretation. Second, all of the subjects in this study were Japanese. Third, in this study, we classified the cases with RVO onset within 3 months as acute, but we could not exclude the possibility of the condition occurring over a year earlier without symptoms. Specifically, the patient cohort included 23 cases of Macular BRVO in the acute group and 17 cases in the recurrent group. In these Macular BRVO cases, the timing of onset may be inaccurate, particularly if macular oedema was absent in the early stages. Fourth, there are various data ranges of aqueous humour cytokine levels, which may be dependent upon individual characteristics. In this study, the volumes of acute and recurrent samples obtained from the same patient were small. We had no choice but to compare the aqueous humour from different patients in the acute and recurrent phases. Thus, it is unclear which cytokines are significant in recurrence and their role in PVD. Fifth, while multiple cytokines were lower in recurrent RVO compared to controls, assessing cytokine changes over time in RVO is subject to selection bias, as longitudinal sampling is ethically limited to cases experiencing recurrence. In addition, this study did not longitudinally measure aqueous humour cytokine levels in the same patients before and after anti-VEGF treatment. Therefore, causal relationships between anti-VEGF therapy and changes in cytokine profiles could not be directly evaluated. Future longitudinal studies measuring aqueous cytokine levels before and after treatment in the same patients are warranted. Furthermore, while patients who underwent PC within four months were excluded from this study, eleven patients had received photocoagulation corresponding to the extent of their non-perfusion area. Patients who had received STTA more than four months prior to the analysis were also included. Although these samples are considered to have been collected after the acute inflammatory phases of both procedures had subsided—as the IL-6 concentrations and other cytokine levels in the STTA group did not show suppressed values, suggesting the anti-inflammatory effect had diminished—we cannot definitively rule out persistent modifying effects from these prior treatments. These effects, such as a reduction in retinal oxygen demand due to PC, might have biased the pathology toward improvement and potentially influenced the cytokine data. Finally, in this study, data from both eyes of the same patient were used as independent data in the recurrent CRVO group. Although the statistical impact on the overall cohort is minimal, this may potentially influence the results.

As described in the Methods section, this study was designed as a hypothesis-generating rather than a hypothesis-testing investigation. Therefore, further well-designed studies are required to confirm and extend our findings.

## Conclusion

Inflammatory cytokine profiles in recurrent retinal vein occlusion differ between branch and central RVO and are characterized by reduced levels of multiple inflammatory cytokines compared with control eyes. These findings highlight distinct inflammatory features of recurrent RVO and provide a basis for future studies investigating disease progression and treatment resistance in RVO.

## Supplementary Information

Below is the link to the electronic supplementary material.

Supplementary Material 1 (15.7 KB)

## Data Availability

The datasets used and/or analysed in the present study are available from the corresponding author on reasonable request.
