# Supplementary material for: Aqueous humour cytokine profiles in retinal vein occlusion: a comparative study of acute, chronic recurrent, and control eyes
Source: Graefes Arch Clin Exp Ophthalmol. 2026 Feb 21;264(6):1833–42. doi: 10.1007/s00417-026-07152-0 (PMC13197360; doi:10.1007/s00417-026-07152-0)
Supplement: Supplementary file 1 — Supplementary Material 1 (15.7 KB) [file 417_2026_7152_MOESM1_ESM.docx]

**Supplementary Table 1. Biological role of each cytokine.**

| pro-inflammatory | anti-inflammatory | Chemotactic Chemokines |
| --- | --- | --- |
| IL-6, VEGF | IL-10 | IP-10, MCP-1, CCL11, CXCL1, CXCL12, CXCL13, |

IL-6, interleukin -6; IL-10, interleukin; IP-10, interferon-γ-inducible protein 10; VEGF, vascular endothelial growth factor; MCP-1, monocyte chemoattractant protein-1;CXCL1, C-X-C motif chemokine ligand 1; CXCL12, C-X-C motif chemokine ligand 12; CXCL13, C-X-C motif chemokine ligand 13; CCL11, C-C motif chemokine ligand 11
